# Plant-based dietary patterns are associated with lower body weight, BMI and waist circumference in older Australian women

**DOI:** 10.1017/S1368980021003852

**Published:** 2022-01

**Authors:** Jessica JA Ferguson, Christopher Oldmeadow, Gita D Mishra, Manohar L Garg

**Affiliations:** 1 Nutraceuticals Research Program, School of Biomedical Sciences & Pharmacy, University of Newcastle, 305C Medical Science Building, Callaghan, NSW 2308, Australia; 2 Hunter Medical Research Institute, University of Newcastle, New Lambton, NSW, Australia; 3 Clinical Research Design, Information Technology and Statistical Support Unit, Hunter Medical Research Institute, University of Newcastle, New Lambton, NSW, Australia; 4 School of Public Health, The University of Queensland, QLD, Australia

**Keywords:** Plant-based diets, Overweight, Obesity, Women, Dietary patterns

## Abstract

**Objective:**

To investigate the association between plant-based diets (PBD) and overweight/obesity compared to regular meat eaters in older women.

**Design:**

Cross-sectional analysis.

**Setting:**

1946–1951 birth cohort of the Australian Longitudinal Study on Women’s Health (ALSWH). PBD were categorised as vegan, lacto-ovo vegetarian, pesco-vegetarian, semi-vegetarian and regular meat eaters. Outcomes included body weight (BW), BMI and waist circumference (WC).

**Participants:**

Women who completed Survey 7 (*n* 9102) with complete FFQ data.

**Results:**

Compared to regular meat eaters, BW, BMI and WC were significantly lower in pesco-vegetarians (−10·2 kg (95 % CI −5·1, −15·2); −3·8 kg/m^2^ (95 % CI −2·0, −5·6); −8·4 cm (95 % CI −3·9, −12·9)) and BW and BMI lower in lacto-ovo vegetarians (−7·4 kg (95 % CI −1·2, −13·6); −2·9 kg/m^2^ (95 % CI −0·6, −5·1)). In regular meat eaters, individuals consuming meat daily or multiple times/d had significantly higher BW, BMI and WC compared to those consuming meat >2 times/week but <daily or multiple times/d (2·5 kg (95 % CI 1·5, 3·5); 0·9 kg/m^2^ (95 % CI 0·5, 1·3) and 2·2 cm (95 % CI 1·3, 3·1)) and those consuming meat >1 but ≤2 times/week (6·8 kg (95 % CI 1·8, 11·8); 2·1 kg/m^2^ (95 % CI 0·3, 4·0) and 6·0 cm (95 % CI 1·7, 10·4)). This association was dose-dependent such that for every increase in category of weekly meat intake (i.e. >1 time/week but ≤2 times/week; >2 times/week but less than daily, and daily or multiple times/d), an associated 2·6 kg (95 % CI 1·8, 3·4) increase in BW, 0·9 kg/m^2^ (95 % CI 0·6, 1·2) increase in BMI and 2·3 cm (95 % CI 1·6, 3·0) increase in WC was reported.

**Conclusions:**

BW, BMI and WC are lower in women following PBD and positively associated with increasing meat consumption. Results were robust to adjustment for confounders including physical activity levels, smoking status, habitual alcohol intake, use of supplements, and hormone replacement therapy.

In Australia, overweight and obesity was the leading modifiable risk factor contributing to non-fatal burden of disease in 2015^(1)^. Overweight and obesity contributed to 45 % of the burden from endocrine disorders, 36 % of the burden from kidney and urinary diseases and 19 % of the CVD burden^(1)^. Two in every three Australians over the age of 18 years are overweight or obese, with higher rates of obesity amongst older adults such that only 16 % of adults aged 18–24 were obese compared to 41 % of adults aged 65–74 in 2017–2018^(2)^. Waist circumference (WC) mainly reflects abdominal fat storage (central obesity) and according to the WHO, is positively associated with chronic disease risk^(3)^. In Australia, the proportion of adults with a WC associated with a substantially increased risk of chronic conditions was higher in women (46 %) than men (36 %), with risk increasing with age. These findings are similar to the USA with 59 % of adults in 2015–2016 having abdominal obesity as measured by WC^(4)^. Several factors can contribute to overweight and obesity, with one of the most modifiable factors being diet and lifestyle. It is well established that inadequate fruit and vegetable consumption is a risk factor for overweight and obesity as well as other non-communicable diseases such as type 2 diabetes, CVD and chronic kidney disease^(5,6)^. Moreover, it has been reported that higher intakes of meat have been positively associated with BMI, WC, obesity, and central obesity^(7)^. Amongst all the major food groups, meat availability is most highly correlated with prevalence of obesity, overweight and mean BMI even after adjusting for total caloric availability and physical inactivity^(8)^.

The switch to plant-based diets (PBD) is not only an emerging societal trend but a global movement due to various reasons such as perceived healthiness^(9,10)^, ethical/moral concerns^(9,11)^, improved sustainability of the food system and reduced environmental impact^(12–14)^. Interestingly, Pribis *et al.* reported that younger individuals under the age of 20 years are more likely to choose a vegetarian diet pattern for ethical/moral and environmental reasons, while health reasons drive this dietary choice in middle-aged individuals (41–60 years)^(9)^. Diets with an emphasis on higher intakes of plant foods and lower intakes of animal foods have been coined the umbrella term ‘plant-based’, encompassing a range of diet patterns with the most commonly known being vegan (nil animal products), lacto-vegetarian (including dairy products), lacto-ovo vegetarian (including dairy products and eggs) through pesco-vegetarian (including fish and seafood with/without dairy and eggs) and semi-vegetarian (very minimal and/or infrequent consumption of meat). PBD have been shown to be associated with lower risk of cardiovascular morbidity and mortality^(15)^, type 2 diabetes mellitus^(16)^, high blood pressure^(17)^, high cholesterol^(18)^ and overweight/obesity^(16,19)^.

The most recent Australian-based cohort study in 2017 by Mihrshahi *et al.* found no difference in all-cause mortality between vegetarians and non-vegetarians, however, compared with regular meat eaters, ‘complete vegetarians’ (defined by the authors as consuming nil animal flesh) were less likely to be overweight/obese or have cardio-metabolic diseases such as hypertension, stroke, heart disease and type 2 diabetes^(20)^. Complete vegetarians also tended to be females and had healthier lifestyle behaviours such as lower prevalence of smoking and risky alcohol consumption. Noteworthy, this study had a small prevalence of vegetarians and determination of ‘vegetarian’ status was undertaken using only short questions that referred to meat consumption and excluded foods from participant’s diets. A food frequency questionnaire (FFQ) was not employed, thereby minimising the breadth of dietary pattern categorisation and moreover, vegan and lacto-ovo vegetarian diets were unable to be distinguished in this cohort^(20)^.

Given the higher prevalence of obesity in older Australian adults and PBD are more commonly followed by women^(20,21)^, the primary aim of this study is to investigate the association between PBD, and overweight/obesity compared to regular meat eaters in a large Australian population-based cohort of older women. The secondary aim of this study is to investigate whether there is a relationship between the frequency of meat intake and overweight/obesity amongst regular meat eaters.

## Methods

### Study population and setting

This study included participants from the Australian Longitudinal Study on Women’s Health (ALSWH). ALSWH was established in 1996 as a result of an Australian government initiative to investigate the health and well-being of Australian women. Women were randomly selected from the Australian Medicare database which covers all citizens and permanent residents including immigrants and refugees^(22)^. This national population-based study selected women born in the following birth cohorts: 1921–1926, 1946–1951 and 1973–1978. The total baseline sample recruited over 40 000 women and was demonstrated to be a representative sample^(23)^. Women were surveyed using self-administered questionnaires in 1996 (Survey 1), 1998 (Survey 2) and every 3 years thereafter until 2016 (Survey 8). Full details of recruitment and response rates for all surveys have been reported elsewhere^(23,24)^.

**Fig. 1:**
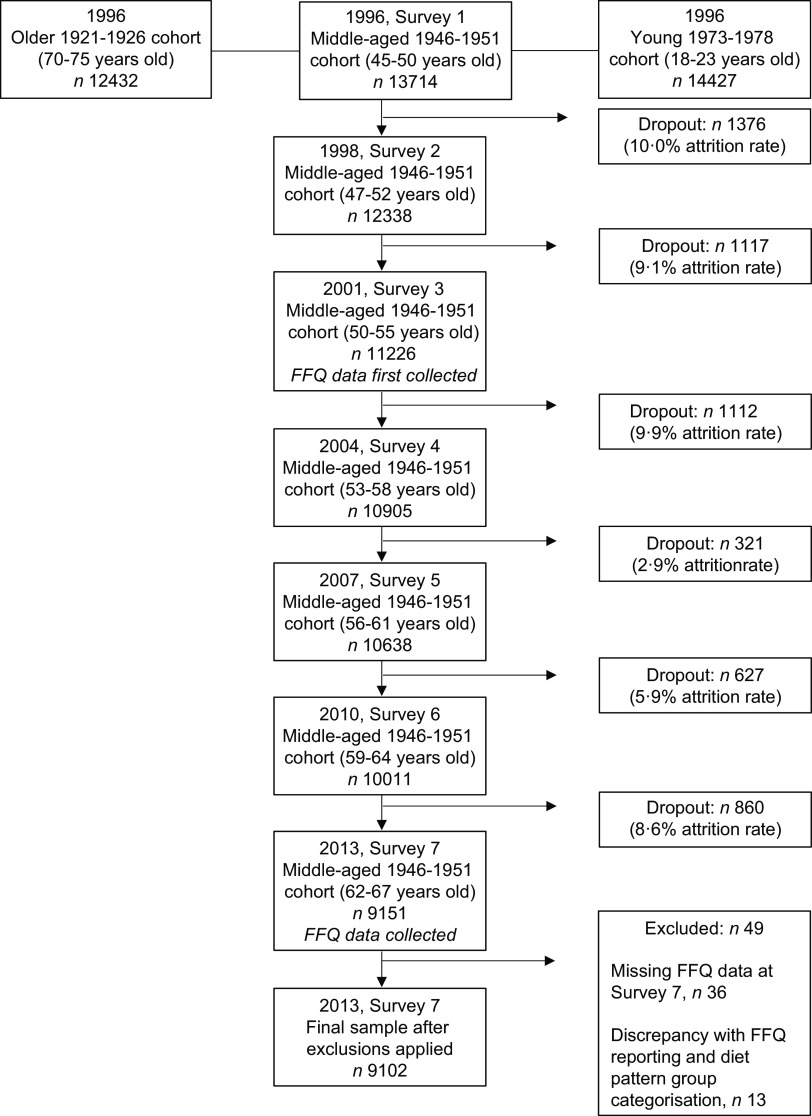
Australian longitudinal study of women’s health: flowchart of the 1946–1951 cohort subjects

The present study examined data from the 1946–1951 cohort of the ALSWH collected in 2013 (Survey 7) as well as FFQ data conducted as part of Survey 7. FFQ data were only collected at Survey 3 (year 2001) and Survey 7 (year 2013), and thus Survey 7 was used as this is the most recent dietary data collected in this birth cohort. The current analysis examines body weight (BW), BMI and WC in all women with dietary data available. Women with missing dietary data at Survey 7 were excluded from analysis in the present study (Fig. 1). Demographic and health behavioural measures such as area of residence, smoking status, alcohol intake and physical activity were also included as part of the survey.

### Dietary assessment

The Dietary Questionnaire for Epidemiological Studies (DQES) version 2 was included as part of Survey 7. The DQES reports usual intake of seventy-four foods and beverages over the previous 12 months using a 10-point frequency scale with responses ranging from ‘never’ to ‘3 or more times/d’. Photographs of portion sizes for meat, vegetables and casseroles were also included. Usual consumption of six different alcoholic beverages was also included as part of the FFQ including two questions assessing the amount of alcohol consumed on days when respondents drank using a 10-point scale ranging from ‘one’ to ‘ten or more’. Additional questions were asked about types of foods consumed such as bread, dairy products, and fat spreads and total amount of daily servings consumed for vegetables, fruit, bread, dairy products, eggs, fat spreads and added sugars. The development and validation of the DQES have been reported previously in a sample of Australian women using weighed food records^(25)^.

### Dietary pattern categorisation

Different categories of PBD and regular meat eaters were classified according to a previous longitudinal cohort study (‘45 and Up Study’) conducted by the Sax Institute which consisted of over 250 000 Australian adults aged 45 years or older^(20)^. In the present study, responses from the DQES for respective food (meat, fish, dairy products, eggs, fat spreads) and beverage (dairy products) intake were used to define PBD and regular meat eaters. No distinction between meat types were made for categorising regular meat eaters. Responses from the DQES were converted to weekly equivalents by assigning scores to each frequency category, with ‘1 time/week’ receiving a score of one, and the remaining responses calculated as a factor of one. For example, ‘Less than once per month’ received a score of 0·15, ‘1–3 times/week’ a score of 0·5, ‘2 times/week’ a score of 2 etc. The ‘45 and Up Study’ did not distinguish vegan or lacto-ovo vegetarians because only brief questions were used to capture dietary data, not a 24-h recall or FFQ. In the present study, vegans were classified as those who reported excluding all animal flesh and animal-derived foods such as dairy products and eggs, and lacto-ovo vegetarians classified as those eating nil beef, lamb, pork, chicken, turkey, duck, processed meat, fish or seafood and consumed animal-derived foods such as dairy products and/or eggs at least 1 time/week or more (Table 1).

**Table 1:** Classification of diet groups by the number of time(s) foods were consumed/week*

	Vegan (*n* 8)	Lacto-ovo vegetarian (*n* 48)	Semi-vegetarian (*n* 45)	Peso-vegetarian (*n* 74)	Regular meat eater (*n* 8927)
Number of time(s)/week consumed:					
Beef, veal, chicken, lamb, pork, bacon, ham, corned beef, luncheon meats or salami, sausages or frankfurters	0	0	≤1	0	0 or≥1
Fish, steamed, grilled, or baked; fish, fried (include take-away), fish, tinned (salmon, tuna, sardines etc.)	0	0	0 or≤1	≥1	0 or≥1
Total of above categories	0	0	≤1	≥1	>1
Usual eating habits†:					
Milk, cheese, ice-cream, yoghurt	Nil	Y	N/A	N/A	N/A
Butter, butter and margarine blends	Nil	Y	N/A	N/A	N/A
Eggs	Nil	Y	N/A	N/A	N/A

N/A is not used for coding into diet groups.

* DQES items were converted to weekly equivalents by assigning scores to each frequency category. With ‘1 time/week’ receiving a score of 1, and the remaining responses calculated as a factor of 1.

† Only habitual intake (and not frequency) of these foods was required for classification of vegan and lacto-ovo vegetarian. Frequency on intake was not provided for butter and butter and margarine blends in the DQES.

### Frequency of meat and fish intake

The frequency of meat intake was defined across four categories: never i.e. never eat meat; ≤1 time/week; >1 time/week but ≤2 times/week; >2 times/week but less than daily and lastly, daily or multiple times/d. The same categorisation process was used for the frequency of fish intake.

### Anthropometric outcomes

Outcomes of interest included self-reported weight (kg), BMI (kg/m^2^) and WC (cm). In Survey 7, participants were instructed to report height and BW with no clothes or shoes. Participants were also instructed to measure WC to the nearest centimetre whilst wearing only underwear, locating the waistline at the navel with the ability to fit their little finger comfortably under the tape to ensure it was not too tight. Self-reported BMI data from the ALSWH have been previously validated^(26)^.

### Covariates

At every survey, participants were asked questions regarding demographic factors and health and lifestyle behaviours. Covariates relevant to the current study include physical activity level, smoking status, habitual alcohol intake, risky alcohol drinking behaviour, use of hormone replacement therapy and use of supplements known to potentially influence weight, BMI or WC i.e. Vitamin D, fish oils and coenzyme Q10 (CoQ10). Physical activity levels were derived from previously validated questions^(27)^ regarding duration of certain types of physical activities in the last week such as walking, moderate and vigorous intensity. Smoking status has been condensed and frequency summarised as ‘not at all’, ‘less than weekly’, ‘weekly’ and ‘daily’. The frequency of alcohol intake was condensed and summarised as ‘never’, ‘1–4 drinks/d’ and ‘≥5 drinks/d’. Alcohol drinking behaviour was derived as per the National Health and Medical Research Council’s alcohol guidelines with the following classifications: ‘non-drinker’, ‘rarely drinks’, ‘low-risk drinker’ (≤2 standard drinks/d), ‘risky drinker’ (3–4 standard drinks/d) and ‘high-risk drinker’ (≥5 standard drinks/d)^(28)^.

### Statistical analyses

Statistical analysis was conducted using StataCorp 2016 (Stata Statistical Software: Release 14.2 StataCorp. LP). The distributions of continuous variables were inspected using histograms and summarised as mean ± sD for symmetric distributions or median and IQR for skewed distributions. Prevalence of the levels of categorical variables are presented as frequency (*n*) and percentage (%). For comparison of skewed continuous variables, Kruskall–Wallis was used and one-way ANOVA for symmetrically distributed continuous variables with Tukey’s post hoc analysis to compare differences in anthropometric measures between the frequency of meat/fish categories within each respective dietary pattern group. Differences in proportions for categorical data were compared using chi-square or Fisher’s exact test. Separate linear regression models were used to examine the crude association between the frequency of meat/fish intake (as independent variables) and BW, BMI and WC (as dependent variables). A collapsed dietary pattern group consisting of ‘semi-vegetarian’ + ‘regular meat eaters’ to represent ‘all meat eaters’ was used for further exploration in linear regression. Multiple linear regression was performed to adjust for potential confounding factors including physical activity levels, smoking status, habitual alcohol intake, use of supplements and hormone replacement therapy. For adjusted analyses, multiple imputation was used to account for missing data using the chained regression equations method, with results from 10 imputed datasets pooled using Rubin’s method. Regression coefficients (or mean differences) and 95 % confidence intervals were reported for relevant tests.

## Results

### Characteristics of study population

A total of forty-nine participants were unable to be included in the analyses as thirty-six of these had incomplete FFQ data and thirteen had discrepancies between responses for frequency of meat consumption and amount of steak portion consumed i.e. when asked ‘*Over the last 12 months, on average, how often did you eat the following foods?*’; individuals who answered ‘*never*’ for all meat fields, yet also had a response for consuming a specific portion size of steak to the question ‘When you ate steak, did you usually eat’ were excluded based on not being able to confidently categorise their meat-eating status (Fig. 1). Therefore, a total of 9102 participants were included for analyses in the current study. Dietary pattern categorisation revealed four PBD groups: vegan (*n* 8, 0·1 %), lacto-ovo vegetarian (*n* 48, 0·53 %), pesco-vegetarian (*n* 74, 0·81 %), semi-vegetarian (*n* 45, 0·49 %) and regular meat eaters (*n* 8927, 98·1 %) as the fifth dietary pattern group (Table 1). In the total sample population, women had a mean age of 64·3 (sd 1·5) years and most lived in either major cities or regional areas, were non-smokers and on hormone replacement therapy. It has been previously reported that most women from the 1946–1951 cohort were born in Australia^(24)^. Only a small proportion (6 %) of women engaged in risky or high-risk alcohol drinking behaviour, 47 % of women were taking fish oil supplements, over a third taking Vitamin D supplements and 5 % taking CoQ10 supplements. Nearly 64 % of the sample were overweight/obese and overall, women weighed on average 73·7 (sd 15·6) kg which was classed as overweight with a BMI of 27·8 (sd 5·7) kg/m^2^ and a WC of 91·3 (sd 13·7) cm (Table 2).

**Table 2:** Characteristics of subjects across diet categories at Survey 7 in the Australian longitudinal study on women’s health 1946–1951 cohort. All values are presented as counts and percentages in parentheses unless otherwise specified

	Vegan (*n* 8)			Lacto-ovo vegetarian (*n* 48)			Pesco-vegetarian (*n* 74)			Semi-vegetarian (*n* 45)			Regular meat eater (*n* 8927)			Total sample (*n* 9102)			*P* *
	Mean	sd	*n* †	Mean	sd	*n*	Mean	sd	*n*	Mean	sd	*n*	Mean	sd	*n*	Mean	sd	*n*	
Age (years)	64·4	1·8		64·1	1·5		64·3	1·5		64·6	1·5		64·3	1·5		64·3	1·5		0·78
Height (cm)	162·4	6·7		162·6	5·4		162·8	7·1		162·9	6·7		162·9	6·6	8916	162·9	6·6	9091	1·00
Weight (kg)	63·7	9·7		66·4	15·3	47	63·7	13·4	72	71·0	13·4	43	73·8	15·6	8629	73·7	15·6	8799	<0·001
WC (cm)	79·9	12·6	7	87·3	13·7	45	83·0	11·8	68	89·0	13·2	38	91·4	13·7	7980	91·3	13·7	8138	<0·001
BMI (kg/m^2^)	24·1	3·1		25·0	5·0	47	24·0	4·5	72	26·8	5·3	43	27·8	5·7	8620	27·8	5·7	8790	<0·001
Overweight or obese‡	2	25·0		21	44·7		25	34·7		27	62·8		5536	64·2		5611	63·8		<0·001
Residence§																			
Major cities	5	62·5		23	47·92		35	47·3		19	43·18		3408	38·29		3490	38·5		<0·008
Inner regional	3	37·5		23	47·92		30	40·54		12	27·27		3541	39·79		3609	39·8		
Outer regional	0	0		1	2·08		8	10·81		11	25·00		1697	19·07		1717	18·9		
Remote	0	0		1	2·08		1	1·35		2	4·55		254	2·85		258	2·8		
Retired/never worked	4	57·1		21	43·8		37	50·0		29	65·9		5158	58·8		5249	58·7		0·096
Smoking																			0·22
Not at all	8	100		46	97·9		69	94·5		40	89·9		8271	93·2		8434	93·2		
< Weekly	–			1	2·13		1	1·37		1	2·22		43	0·48		46	0·5		
Weekly	–			–			–			–			37	0·4		37	0·4		
Daily	–			–			3	4·1		4	8·9		522	5·9		529	5·8		
Alcohol intake																			<0·001
Never	5	62·5		14	29·8		22	29·7		22	53·7		1411	16·3		1474	16·7		
1–4 drinks/d	3	37·5		33	70·2		52	70·3		19	46·3		7156	82·7		7263	82·3		
≥5 drinks/d	–			–			–			–			86	1·0		86	1·0		
Risky/high-risk alcohol drinker‖	0	0		3	6·25		2	2·7		1	2·4		525	6·0		531	6·0		<0·0001
Supplement use¶																			
Fish oils	1	14·3		11	23·9		42	57·5		13	29·5		4162	47·2		4229	47·1		<0·001
Vitamin D	1	12·5		18	39·1		33	45·2		13	30·2		3074	34·9		3139	35·0		0·22
CoQ10	1	14·3		6	13·3		9	12·3		5	11·4		443	5·1		464	5·2		0·001
HRT	0	0		2	4·2		10	3·5		2	4·4		832	9·3		8234	90·7		0·36
Physical activity (min/week)**	Median	IQR	*n*	Median	IQR	*n*	Median	IQR	*n*	Median	IQR	*n*	Median	IQR	*n*	Median	IQR	*n*	
Brisk walk	180	210		165	345		180	210	73	75	240	42	120	270	8675	120	270	8846	0·062
Moderate	15	150		30	130		0	90	73	0	30	42	0	120	8735	0	120	8933	0·19
Vigorous	150	240		125	270		120	300	73	60	125	41	150	300	8659	150	300	8829	0·042

CoQ10, coenzyme Q10; HRT, hormone replacement therapy; IQR, interquartile range; WC, waist circumference.

* *P*-value represents difference across groups. Normally distributed continuous data compared using ANOVA, non-normally continuous compared using Kruskall–Wallis, categorical data compared using Fisher’s Exact. Data for residence area and risky/high-risk alcohol drinker compared using multinomial logistic regression.

† For measures with missing data that are not already presented as counts and percentages, the number of participants has been provided.

‡ Overweight or obese defined by the WHO as overweight, BMI > 25 kg/m^2^ and < 30 kg/m^2^; Obese ≥ 30 kg/m^2^.

§ Measure of remoteness according to the 2001 Australian Standard Geographical Classification by the Australian Bureau of Statistics.

‖ National Health and Medical Research Council alcohol status.

¶ Dietary supplement use in the past 4 weeks.

** The amount of time (minutes) spent undertaking each type of activity in the last week.

### BW, BMI and WC across dietary pattern categories

Individuals following a vegan and pesco-vegetarian dietary pattern had the lowest BW and BMI followed by lacto-ovo vegetarians then semi-vegetarians. Compared to regular meat eaters, BW and BMI was statistically significantly lower in lacto-ovo vegetarians (−7·4 kg (95 % CI −1·2, −13·6) and −2·9 kg/m^2^ (95 % CI −0·6, −5·1)) and pesco-vegetarians (−10·2 kg (95 % CI −5·1, −15·2) and −3·8 kg/m^2^ (95 % CI −2·0, −5·6)). A similar trend was reported for WC, with individuals following a vegan dietary pattern having the lowest WC followed by pesco-vegetarians, lacto-ovo vegetarians through to semi-vegetarians. Compared to regular meat eaters, only pesco-vegetarians had a significantly lower WC (−8·4 cm (95 % CI −3·9, −12·9)).

### Lifestyle characteristics across diet groups

At least half of the women following PBD resided in major cities and the highest proportion of regular meat eaters resided in outer regional areas followed by major cities (Table 2). Alcohol intake/week and engagement in risky or high-risk alcohol drinking behaviour differed across the dietary pattern groups whereby regular meat eaters and lacto-ovo vegetarians had a higher proportion of women who partake in risky or high-risk alcohol drinking behaviours. Average minutes of physical activity undertaken/week was mostly similar across groups except for vigorous physical activity where semi-vegetarians engaged in the least amount of vigorous physical activity and vegans and regular meat eaters engaged in the most. Use of fish oil supplements significantly differed across the groups with pesco-vegetarians having the highest prevalence of use followed by regular meat eaters, semi-vegetarians, lacto-ovo vegetarians through to vegans. Those following PBD tended to be more likely to use CoQ10 supplements compared to regular meat eaters (∼11–14 % *v*. 5 %, respectively). Smoking status, hormone replacement therapy and Vitamin D supplement use were similar across all groups.

### BW, BMI and WC across frequency of meat and fish intake in meat- and fish-eating dietary pattern groups

BW, BMI and WC differed significantly across the frequency of meat intake categories in individuals who were regular meat eaters. Individuals who consumed meat daily or multiple times/d had a significantly higher BW, BMI, and WC compared to those who consumed meat more than 2 times/week (but less than daily or multiple times/d) (2·5 kg (95 % CI 1·5, 3·5); 0·9 kg/m^2^ (95 % CI 0·5, 1·3) and 2·2 cm (95 % CI 1·3, 3·1)) and compared to those that consumed meat >1 but≤2 times/week (6·8 kg (95 % CI 1·8, 11·8); 2·1 kg/m^2^ (95 % CI 0·3, 4·0) and 6·0 cm (95 % CI 1·7, 10·4)) (Table 3). BW, BMI, and WC did not differ statistically across the frequency of fish intake in pesco-vegetarians. In regular meat eaters, a significant difference across the frequency of fish intake was observed for BW (*P* = 0·0358), BMI (*P* = 0·0062) and WC (*P* = 0·0109). Regular meat eaters who consumed fish more than 2 times/week (but less than daily or multiple times/d) had significantly lower BW (−3·4 kg (95 % CI −6·8, −0·02)) than those who did not consume fish as part of their regular diet. The same trend was observed for BMI; however, statistical significance was lost after performing post hoc comparisons (*P* = 0·054). WC was significantly lower in regular meat eaters who consumed fish more than 2 times/week (but less than daily or multiple times/d) compared to those who only consumed fish≤1 time/week (−1·3 cm (95 % CI −2·5, −0·2)).

**Table 3:** Weight status by frequency categories of meat and fish intake/week across meat- and fish-eating diet categories (pesco-vegetarian, semi-vegetarian and regular meat eater) in the Australian longitudinal study on women’s health 1946–1951 cohort

	Pesco-vegetarian								Semi-vegetarian				Regular meat eater									
Categories of meat and fish intake frequency/week*																						
	1		2		3		4		0		1		0		1		2		3		4	
	Mean	sd	Mean	sd	Mean	sd	Mean	sd	Mean	sd	Mean	sd	Mean	sd	Mean	sd	Mean	sd	Mean	sd	Mean	sd
Wt (kg)																						
Meat	–	–	–	–	–	–	–	–	–	–	71·0	13·4	–	–	–	–	67·5^a^	12·0	71·8^a^	15·6	74·3^b^	15·6
*n*	–		–		–		–		–		43		–		–		54		1636		6439	
Fish	65·7	15·3	65·1	15·3	61·6	12·5	67·1	15·9	72·2	16·9	70·7	12·6	76·9^a^	17·7	74·3^a,b^	16·0	73·9^a,b^	15·8	73·5^b^	15·1	74·3^a,b^	16·2
*n*	3		18		38		13		9		34		163		1578		2530		3946		412	
BMI (kg/m^2^)																						
Meat	–	–	–	–	–	–	–	–	–	–	26·8	5·3	–	–	–	–	25·9^a^	5·1	27·1^a^	5·8	28·0^b^	5·7
*n*	–		–		–		–				43		–		–		53		1634		6933	
Fish	23·7	5·1	24·9	4·5	23·3	4·1	25·1	5·6	28·4	6·2	26·4	5·0	28·9	6·9	28·1	5·9	27·8	5·8	27·7	5·5	28·2	5·9
*n*	3		18		38		13		9		34		163		1574		2529		3944		410	
WC (cm)																						
Meat	–	–	–	–	–	–	–	–	–	–	89·0	13·2	–	–	–	–	85·8^a^	16·6	89·7^a^	13·5	91·8^b^	13·6
*n*	–		–		–		–		–		38		–		–		55		1517		6408	
Fish	84·8	8·7	83·8	12·2	82·9	12·0	81·7	12·8	95·0	13·2	87·7	13·1	93·2^a,b^	16·6	92·2^a^	14·2	91·5^a,b^	13·2	90·9^b^	13·5	90·8^a,b^	14·9
*n*	3		17		36		12		7		31		145		1448		2356		3668		363	

WC, waist circumference; Wt, weight.

* Frequency of dietary meat and fish intake are defined as follows: 1, ≤1 time/week; 2, >1 to 2 times/week; 3, >2 times/week; 4, daily or multiple times/d. Characteristics were compared across frequencies of intake within each diet group using ANOVA and Tukey’s post hoc analysis. Dashes in cells represent non-applicability due to dietary group definition.

^a,b^Mean values within a row with unlike superscript letters are significantly different (*P* < 0·05).

### Association between BW, BMI and WC and frequency of meat and fish intake in meat- and fish-eating dietary pattern groups

**Fig. 2:**
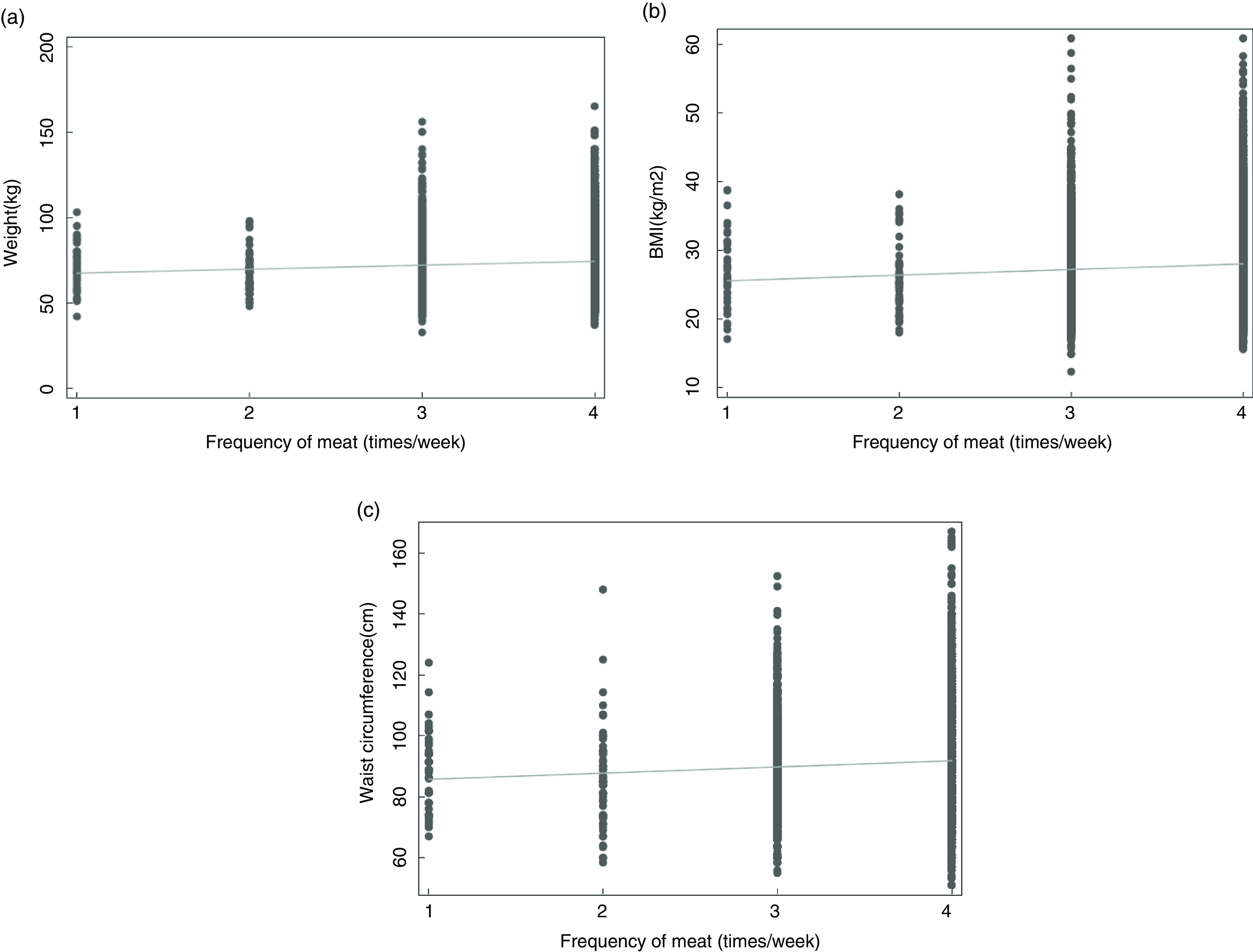
Association between the frequency of weekly meat intake and body weight (a), BMI (b) and waist circumference (c) in all meat eaters. Fitted values are represented by the line. The frequency of meat intake categories: 1, ≤1 time/week; 2, >1 to 2 times/week; 3, >2 times/week; 4, daily or multiple times/d

In regular meat ears, BW, BMI and WC were positively associated with incremental increases in categories of weekly frequency of meat intake. For example, women who ate meat >2 times/week were 2·6 kg heavier than women who only ate meat >1 to 2 times/week (Table 4). The same association was observed in all meat eaters (combined semi-vegetarian + regular meat eaters) (Fig. 2). The associations between frequency of meat intake/week and BW, BMI and WC remained after adjusting for confounders with minor changes in coefficient size for regular meat eaters (BW = −3·6 %, BMI = 1·3 %, WC = −2·4 %) and all meat eaters (BW = 0·7 %, BMI = 5·8 %, WC = −2·1 %) Crude and adjusted means have been reported in Supplemental Table 1. There was no statistically significant association between BW or BMI and frequency of fish intake in pesco-vegetarians, however, increasing fish intake was associated with a small but significantly lower in regular meat eaters and all meat eaters. The associations for BW and WC remained after adjusting for confounders with small reductions in coefficient size (−6·9 % and −14·8 %, respectively) for regular meat eaters and all meat eaters (−8·5 % and −15·0 %, respectively), however, significance was lost for BMI in both regular meat eaters (95 % CI −0·26, 0·01) and all meat eaters (95 % CI −0·25, 0·03). When weekly meat intake was added to the model, greater reductions in BW (−0·80 kg (95 % CI −1·19, −0·40)), BMI (−0·28 kg/m^2^ (95 % CI −0·42, −0·13)) and WC (−0·91 cm (95 % CI −1·26, −0·55)) were observed in regular meat eaters and all meat eaters (−0·80 kg (95 % CI −1·19, −0·40); −0·29 kg/m^2^ (95 % CI −0·42, −0·13) and −0·91 cm (95 % CI −1·27, −0·55)).

**Table 4:** Crude and adjusted associations between the frequency of meat and fish intake categories and anthropometric outcomes in meat-eating diet groups in the Australian longitudinal study on women’s health 1946–1951 cohort. Data are presented as *β* coefficients, standard errors and 95 % confidence intervals

	Total sample			Regular meat eaters			All meat eaters†			Pesco-vegetarian		
	*β*	se	95 % CI	*β*	se	95 % CI	*β*	se	95 % CI	*β*	se	95 % CI
Crude associations–frequency of meat intake												
Weight (kg)												
	2·41***	0·30	1·83, 3·00	2·62***	0·40	1·84, 3·41	2·33***	0·36	1·62, 3·04	n/a	n/a	n/a
BMI (kg/m^2^)												
	0·87***	0·11	0·66, 1·08	0·92***	0·15	0·63, 1·21	0·82***	0·13	0·56, 1·08	n/a	n/a	n/a
WC (cm)												
	1·95***	0·27	1·42, 2·48	2·29***	0·36	1·58, 3·00	2·04***	0·33	1·39, 2·69	n/a	n/a	n/a
Adjusted associations‡–frequency of meat intake												
Weight (kg)												
	2·37***	0·31	1·77, 2·97	2·53***	0·41	1·73, 3·33	2·33***	0·37	1·61, 3·06	n/a	n/a	n/a
BMI (kg/m^2^)												
	0·89***	0·11	0·67, 1·11	0·94***	0·15	0·64, 1·23	0·87***	0·14	0·60, 1·13	n/a	n/a	n/a
WC (cm)												
	1·95***	0·28	1·40, 2·50	2·24***	0·37	1·50, 2·97	2·00***	0·34	1·32, 2·67	n/a	n/a	n/a
Crude associations–frequency of fish intake												
Weight (kg)												
	−0·28	0·18	−0·63, 0·08	−0·41*	0·19	−0·78, −0·04	−0·38*	0·19	−0·74, −0·01	0·15	2·10	−4·04, 4·34
BMI (kg/m^2^)												
	−0·11	0·07	−0·24, 0·02	−0·16*	0·07	−0·29, −0·02	−0·15*	0·07	−0·28, −0·01	−0·01	0·70	−1·41, 1·39
WC (cm)												
	−0·52**	0·17	−0·84, −0·19	−0·61***	1·73	−0·95, −0·27	−0·59***	0·17	−0·92, −0·25	−1·00	1·90	−4·80, 2·80
Adjusted associations–frequency of fish intake												
Weight (kg)												
	−0·26	0·19	−0·63, 0·10	−0·38*	0·19	−0·76, 0·0009	−0·35	0·19	−0·72, 0·03	0·90	2·27	−3·65, 5·45
BMI (kg/m^2^)												
	−0·08	0·07	−0·22, 0·05	−0·12	0·07	−0·26, 0·01	−0·11	0·07	−0·25, 0·03	0·05	0·78	−1·51, 1·60
WC (cm)												
	−0·44*	0·17	−0·77, −0·11	−0·52**	0·18	−0·87, −0·17	−0·50**	0·18	−0·84, −0·16	−0·74	2·09	−4·92, 3·45

WC, waist circumference.

* *P* < 0·05.

† Semi-vegetarian and Regular meat eater diet categories were combined into one group to represent all individuals who eat meat that is not exclusively fish/seafood.

‡ Multiple linear regression was performed to adjust for potential confounding factors: hormone replacement therapy, habitual alcohol intake, smoking status, physical activity (low, moderate and vigorous), use of supplements such as vitamin D, fish oils and coenzyme Q10. Multiple imputation estimates were used to account for missing values across variables.

** *P* < 0·01.

*** *P* < 0·001.

## Discussion

In this sample of older Australian women, cross-sectional analysis revealed that women who followed a PBD had significantly lower BW, BMI and WC compared to regular meat eaters. Moreover, increasing frequency of meat intake was associated with higher BW, BMI, and WC. This relationship was not apparent for frequency of fish intake in pesco-vegetarians, however, women who had a higher weekly intake of fish as part of their regular meat-eating dietary pattern had significantly lower BW and WC.

The prevalence of PBD and regular meat eaters in this sample are comparable to another large Australian population-based cohort study (‘The 45 and Up Study’), however, compared to cohort studies conducted overseas, the prevalence of PBD appear to be lower. The ‘45 and Up Study’ was conducted in males and females with a wider age group (≥45 years)^(20)^. The same PBD definitions were used and the same prevalence of regular meat eaters was reported; however, the prevalence of pesco-vegetarians was lower and semi-vegetarians higher^(20)^. A Belgium cohort study reported mostly meat eaters (83·3 %), 11·8 % ‘semi-vegetarians’ (almost vegetarians, part-time vegetarians, and pesco-vegetarians) and 1·6 % vegetarians (lacto-ovo vegetarians and vegans)^(29)^. Discrepancies in PBD prevalence across studies could be largely attributed to the diversity in cultural cuisine, different defining characteristics used and methods of categorising specific PBD e.g. participants self-professing their dietary status *v*. researchers categorising dietary patterns based on FFQ results or statistical methodologies such as principal components factor analysis.

Previous cohort studies in men and women have reported that compared with regular meat eaters, individuals consuming PBD were more likely to have a lower BMI^(16,30,31)^. In two of these studies, lacto-ovo vegetarians, pesco-vegetarians and semi-vegetarians had incrementally higher BMI compared to vegans^(16,30)^. As with another larger cohort study, some of the difference in BMI was partly explained when adjusted for non-dietary factors such as smoking status and exercise levels, however, the relationship still remained^(30)^. Interestingly, the pesco-vegetarians in the current study had a comparable BMI to vegans, with incremental increases from lacto-ovo vegetarians to semi-vegetarians through to regular meat eaters. Findings from the current study are clinically relevant in the context of WHO cut-off points for BMI and WC and risk of chronic disease^(3)^. In the current study, women who consumed meat more than once up to 2 times/week had a mean BMI that was in the lower range of the ‘overweight’ (25·00–29·99 kg/m^2^) category and a mean WC classification of ‘increased risk’ (≥80 cm), however, women consuming meat more than 2 times/week and daily or multiple times/d had a mean BMI and WC in the ‘overweight’ and ‘substantially increased risk’ (≥88 cm) categories (respectively). It has been previously reported that increasing meat intake, notably processed and/or red meat intake is associated with higher BW and WC^(7,32–34)^. Higher daily intake of total or animal protein (mainly derived from red/processed meat and poultry; and not plant protein) was associated with yearly weight gain in a European longitudinal cohort study of older adults (54–59 years) and this association was stronger in women^(35)^. Even though regular meat eaters in this sample had a higher proportion of individuals who habitually consume higher amounts of alcohol across the week as well as engage in risky alcohol drinking behaviour compared to PBD individuals, adjustment for alcohol did not affect the associations between meat intake and anthropometric measures. The higher caloric intake including higher dietary fat/saturated fat intake^(7,30)^ and dietary protein^(30,35)^, difference in overall energy balance^(32)^, as well as synergistic effects of unfavourable lifestyle behaviours such as risky alcohol drinking behaviour as reported in the current study and dietary choices associated with meat consumption^(33,34)^; have been argued as plausible reasons for this positive association. Conversely, it has been reported that vegetarians and vegans tend to possess healthier lifestyle behaviours including greater physical activity levels, lower intakes of alcohol and caffeine and lower smoking rates compared to regular meat eaters^(36)^. It has been suggested that dietary protein yielded from higher meat intake may help to maintain and preserve muscle mass in older adults^(37)^, thus focusing on BW and BMI as key outcomes rather than adiposity could be misleading^(32)^. WC may serve as a plausible surrogate marker of adiposity in observational studies, and in this regard the current study along with others^(7,32,33,38,39)^ have reported positive associations between meat intake and WC, which are also consistent with findings published in a recent systematic review and meta-analysis of observational studies^(34)^.

Conversely, women who ate more fish as part of a regular meat-eating dietary pattern had significantly lower BW and WC. Mixed results have been reported with respect to fish intake and associated changes in anthropometry. A large European longitudinal study by Jakobsen *et al.* found no association nor sex-specific differences in findings between fish consumption and associated change in WC after 5·5-year follow-up^(40)^. Findings from the current study are consistent with a previous cohort study by Karlsson *et al.* in middle-aged adults with unspecified dietary patterns whereby an inverse relationship between lean fish intake and WC was reported^(41)^. Noteworthy, the cohort studied by Jakobsen *et al*. had BMI’s and WC’s that were in the healthy range at baseline, whereas the cohort in Karlsson *et al.* and the current study involved individuals who were overweight with elevated WC which could be suggestive of this association only being evident in overweight/obese individuals. A cross-sectional analysis in older Australian adults (65–95 years) reported a sex-specific inverse association between *n*-3 status (a measure of long-term fish intake) and BMI and WC, this relationship remained only in females after adjusting for lifestyle factors^(42)^. Intervention studies have reported significant reductions in weight, BMI, WC and body fat percentage following supplementation with fish/fish oils^(43)^. Greater reductions in BW and BMI have been reported in females compared to males following dietary supplementation with fish oils^(44)^. Collectively, these findings are suggestive of a potential protective effect of a higher fish intake in the context of a regular meat-eating diet for better weight management and lower risk of central obesity. Future studies are warranted to explore the frequency of fish intake in the context of regular meat-eating diets across different categories of BMI and WC to ascertain whether dietary guidelines around fish ought to be specific to BMI or central obesity. Previous studies have reported that pesco-vegetarians (as with other PBDs) lead healthier diet/lifestyle behaviours^(45,46)^, however, interestingly, there was no significant association BW or BMI and frequency of fish intake amongst pesco-vegetarians in this sample. Future studies in Australian women are warranted to examine the relationship between fish intake and BW, BMI and WC to better understand the ideal frequency of fish intake in both the context of a pesco-vegetarian and regular meat-eating dietary pattern for healthy weight management.

Healthful PBD are typically rich in dietary fibre^(30,47)^ and lower in energy density^(48)^ which may underpin the inverse relationship between central obesity and PBD. Dietary fibre has been reported to be associated with less visceral fat^(49)^ with various plausible mechanisms such as increased satiety^(50)^ due to greater food volume without contributing additional absorbable energy^(48)^, reduced glycaemic and insulinemic response to a meal^(51)^ and increased secretion of gastrointestinal satiety hormones^(50)^. In support, low dietary fibre and high protein intake were the strongest dietary factors associated with increasing BMI between and within diet groups (vegans, fish eaters, lacto-ovo vegetarians and meat eaters) in the EPIC-Oxford cohort study^(30)^. Differences in macronutrient intake have been shown to account for about 50 % of the difference in mean BMI between vegans and regular meat eaters^(30)^. Caloric intake has been shown to significantly differ between various PBDs with vegans consuming the least calories^(47)^. Lower BW and BMI associated with long-term adherence to PBD may be linked to lower bone density/mass. A recent systematic review and meta-analysis demonstrated that compared to omnivores, lacto-ovo vegetarians and vegans had significantly lower bone mineral density and vegans had significantly higher fracture risk^(52)^. Tong *et al*. reported that compared to meat eaters, hip fracture risk was higher in vegans followed by pesco-vegetarians and vegetarians, even after adjustment for socio-economic and lifestyle factors including BMI^(53)^. It has been suggested that a lack of essential nutrients required for optimal bone health sourced richly from animal products could be a key contributing factor underpinning these observations^(10)^. Although dietary calcium and total protein are important risk factors for poor bone health, Tong *et al.* reported only slight attenuation in associations between PBD and fracture risk after adjusting for dietary calcium and/or total protein intake, and associations appeared to be stronger without adjustment for BMI^(53)^. A higher BMI may reflect protection against fracture risk due to stronger bones from increased weight-bearing or more cushioning tissue against fall impact for example^(54)^. Others have hypothesised that dietary protein, vitamin B_12_, vitamin D, protein, zinc and *n*-3 fatty acids; which are also primarily sourced from animal products, have a positive impact on bone health and may be lacking in some PBD^(52,55)^. Data on the caloric and macronutrient status across various PBD are scarce and fairly limited by the different classifications of PBD highlighting the importance of appropriately planned PBD to prevent nutritional deficiencies.

We acknowledge several study limitations as these analyses are exploratory within the greater ALSWH. Although the PBD and regular meat eater profiles in this study are somewhat comparable to other population-based cohort studies, it must be acknowledged that the prevalence of the various PBD categories were quite scarce and not large enough to perform meaningful regression analyses. Nonetheless, the authors sought to maintain transparency in reporting these findings, with further exploratory analyses focused on the meat-eating diet groups. Our exposure (diet), outcome measures (BW, BMI, WC) and covariates (i.e. lifestyle factors) relied on self-reported data which has known inherent errors. It is noteworthy, however, that several variables such as FFQ and self-reported anthropometric outcomes have been previously validated. Missing data across the variables explored in this paper also present as a limitation, however, also a common issue with self-reported survey data. The authors tried to account for this by performing additional analyses to impute predicted values for missing data when undertaking regression analyses. Although food group intake was assessed using a validated FFQ, self-reported data are always subject to biases and for this proof-of-concept study we did not explore sub-types of certain food groups such as processed meats, red meats, poultry and fried fish nor were other food groups explored in the current study. Lastly, since the current study is a cross-sectional analysis, causality cannot be confirmed. We present findings from a representative sample of older Australian women and furthermore, the prevalence of overweight/obesity in this cohort (63·8 %) is comparable to the recently reported proportion of overweight/obese Australian women aged 55–64 years (66·6 %; 95 % CI 63·5, 69·7) in 2017–2018^(51)^. To provide consistent translational findings to the Australian population, the authors adopted PBD categories that were used in a previous Australian population-based cohort study consisting of over 260 000 adults by Mihrshahi *et al.*^(20)^. The wide spectrum of dietary assessment and comparative prevalence of overweight/obesity to older Australian women are key strengths of the current study and enhances the overall translational capacity of these findings. To the best of our knowledge, the current study and Mihrshahi *et al.* are the only published works exploring PBD patterns and health outcomes compared to regular meat eaters in the Australian adult population.

## Conclusion

In a representative sample of older Australian women, most ate meat regularly with women following PBD representing a small percentage of the sample. BW, BMI and WC were lower in women following PBD compared to women who were regular meat eaters. Moreover, incrementally higher BW, BMI and WC were associated with increasing frequency of meat intake, however, higher fish intake in women who eat meat was associated with lower BW, BMI and WC. These findings are consistent with international population-based studies, however, contribute to the limited evidence available within the Australian context and support the need for further exploration into the current profile of PBD and associated health implications compared to regular meat eaters in not only women, but men and across the lifespan given the growing emergence of PBD across the globe.

## Supplementary material

For supplementary material accompanying this paper visit https://doi.org/10.1017/S1368980021003852. click here to view supplementary material
