# Supplementary material for: Plant-based dietary patterns are associated with lower body weight, BMI and waist circumference in older Australian women
Source: Public Health Nutr. 2021 Sep 6;25(1):18–31. doi: 10.1017/S1368980021003852 (PMC8825973; doi:10.1017/S1368980021003852)
Supplement: Supplementary file 1 [file S1368980021003852sup.zip › S1368980021003852sup002.docx]

| **Supplementary Table 1.** Crude and adjusted mean values for body weight, BMI and waist circumference across weekly frequency of meat and fish intake in the total sample, regular meat eaters, all meat eaters and pesco-vegetarians. | | |
| --- | --- | --- |
|  | **Crude** | **Adjusted** |
| **Total Sample** | mean±SEM | mean±SEM |
| Weight (kg) |  |  |
| Meat intake category 0^1^ | 64.6±1.1 | 64.0±1.2 |
| Meat intake category 1 | 67.1±0.8 | 66.5±0.9 |
| Meat intake category 2 | 69.5±0.5 | 69.1±0.6 |
| Meat intake category 3 | 71.9±0.3 | 71.6±0.3 |
| Meat intake category 4 | 74.3±0.2 | 74.2±0.2 |
| Fish intake category 0 | 74.3±0.5 | 74.2±0.5 |
| Fish intake category 1 | 74.0±0.3 | 73.9±0.3 |
| Fish intake category 2 | 73.8±0.2 | 73.7±0.2 |
| Fish intake category 3 | 73.5±0.2 | 73.4±0.2 |
| Fish intake category 4 | 73.2±0.3 | 73.1±0.4 |
| BMI (kg/m^2^) |  |  |
| Meat intake category 0 | 24.5±0.4 | 24.1±0.4 |
| Meat intake category 1 | 25.4±0.3 | 25.1±0.3 |
| Meat intake category 2 | 26.2±0.2 | 26.0±0.2 |
| Meat intake category 3 | 27.1±0.1 | 27.0±0.1 |
| Meat intake category 4 | 28.0±0.1 | 27.9±0.1 |
| Fish intake category 0 | 28.0±0.2 | 27.9±0.2 |
| Fish intake category 1 | 27.9±0.1 | 27.8±0.1 |
| Fish intake category 2 | 27.8±0.1 | 27.7±0.1 |
| Fish intake category 3 | 27.7±0.1 | 27.7±0.1 |
| Fish intake category 4 | 27.6±0.1 | 27.6±0.1 |
| Waist circumference (cm) |  |  |
| Meat intake category 0 | 84.0±1.0 | 83.5±1.1 |
| Meat intake category 1 | 85.9±0.8 | 85.6±0.8 |
| Meat intake category 2 | 87.9±0.5 | 87.7±0.5 |
| Meat intake category 3 | 89.8±0.3 | 89.8±0.3 |
| Meat intake category 4 | 91.8±0.2 | 91.8±0.2 |
| Fish intake category 0 | 92.5±0.4 | 92.5±0.4 |
| Fish intake category 1 | 92.0±0.3 | 92.0±0.3 |
| Fish intake category 2 | 91.4±0.2 | 91.5±0.2 |
| Fish intake category 3 | 90.9±0.2 | 91.0±0.2 |
| Fish intake category 4 | 90.4±0.3 | 90.5±0.3 |
| **Regular meat eaters** |  |  |
| Weight (kg) |  |  |
| Meat intake category 2 | 69.1±0.7 | 68.7±0.8 |
| Meat intake category 3 | 71.7±0.4 | 71.5±0.4 |
| Meat intake category 4 | 74.4±0.2 | 74.3±0.2 |
| Fish intake category 0 | 74.8±0.5 | 74.7±0.5 |
| Fish intake category 1 | 74.4±0.3 | 74.3±0.3 |
| Fish intake category 2 | 74.0±0.2 | 73.9±0.2 |
| Fish intake category 3 | 73.6±0.2 | 73.4±0.2 |
| Fish intake category 4 | 73.1±0.4 | 73.0±0.4 |
| BMI (kg/m^2^) |  |  |
| Meat intake category 2 | 26.2±0.3 | 25.9±0.3 |
| Meat intake category 3 | 27.1±0.1 | 26.9±0.1 |
| Meat intake category 4 | 28.0±0.1 | 28.0±0.1 |
| Fish intake category 0 | 28.2±0.2 | 28.1±0.2 |
| Fish intake category 1 | 28.0±0.1 | 27.9±0.1 |
| Fish intake category 2 | 27.9±0.1 | 27.8±0.1 |
| Fish intake category 3 | 27.7±0.1 | 27.7±0.1 |
| Fish intake category 4 | 27.6±0.1 | 27.5±0.1 |
| Waist circumference (cm) |  |  |
| Meat intake category 2 | 87.3±0.7 | 86.9±0.7 |
| Meat intake category 3 | 89.6±0.3 | 89.4±0.3 |
| Meat intake category 4 | 91.9±0.2 | 91.9±0.2 |
| Fish intake category 0 | 92.8±0.4 | 92.9±0.5 |
| Fish intake category 1 | 92.2±0.3 | 92.3±0.3 |
| Fish intake category 2 | 91.6±0.2 | 91.6±0.2 |
| Fish intake category 3 | 91.0±0.2 | 91.0±0.2 |
| Fish intake category 4 | 90.4±0.3 | 90.4±0.3 |
| **All meat eaters^2^** |  |  |
| Weight (kg) |  |  |
| Meat intake category 1 | 67.3±1.0 | 66.5±1.1 |
| Meat intake category 2 | 69.7±0.7 | 69.1±0.7 |
| Meat intake category 3 | 72.0±0.3 | 71.7±0.3 |
| Meat intake category 4 | 74.3±0.2 | 74.2±0.2 |
| Fish intake category 0 | 74.7±0.5 | 74.6±0.5 |
| Fish intake category 1 | 74.3±0.3 | 74.2±0.3 |
| Fish intake category 2 | 74.0±0.2 | 73.8±0.2 |
| Fish intake category 3 | 73.6±0.2 | 73.5±0.2 |
| Fish intake category 4 | 73.2±0.4 | 73.1±0.4 |
| BMI (kg/m^2^) |  |  |
| Meat intake category 1 | 25.5±0.4 | 25.06±0.4 |
| Meat intake category 2 | 26.4±0.2 | 26.03±0.3 |
| Meat intake category 3 | 27.2±0.1 | 27.0±0.1 |
| Meat intake category 4 | 28.0±0.1 | 28.0±0.1 |
| Fish intake category 0 | 28.2±0.2 | 28.0±0.2 |
| Fish intake category 1 | 28.0±0.1 | 27.9±0.1 |
| Fish intake category 2 | 27.9±0.1 | 27.8±0.1 |
| Fish intake category 3 | 27.7±0.1 | 27.7±0.1 |
| Fish intake category 4 | 27.6±0.1 | 27.6±0.1 |
| Waist circumference (cm) |  |  |
| Meat intake category 1 | 85.7±0.9 | 85.1±1.0 |
| Meat intake category 2 | 87.7±0.6 | 87.4±0.6 |
| Meat intake category 3 | 89.8±0.3 | 89.6±0.3 |
| Meat intake category 4 | 91.8±0.2 | 91.9±0.2 |
| Fish intake category 0 | 92.7±0.4 | 92.8±0.4 |
| Fish intake category 1 | 92.2±0.3 | 92.2±0.3 |
| Fish intake category 2 | 91.6±0.2 | 91.6±0.2 |
| Fish intake category 3 | 91.0±0.2 | 91.0±0.2 |
| Fish intake category 4 | 90.4±0.3 | 90.5±0.4 |
| **Pesco-vegetarian** |  |  |
| Weight (kg) |  |  |
| Fish intake category 1 | 63.4±4.2 | 61.7±4.5 |
| Fish intake category 2 | 63.5±2.4 | 62.9±2.5 |
| Fish intake category 3 | 63.7±1.6 | 64.1±1.6 |
| Fish intake category 4 | 63.8±2.9 | 65.4±3.1 |
| BMI (kg/m^2^) |  |  |
| Fish intake category 1 | 24.0±1.4 | 23.8±1.5 |
| Fish intake category 2 | 24.0±0.8 | 24.0±0.9 |
| Fish intake category 3 | 24.0±0.5 | 24.1±0.6 |
| Fish intake category 4 | 24.0±1.0 | 24.3±1.1 |
| Waist circumference (cm) |  |  |
| Fish intake category 1 | 84.8±3.8 | 86.1±4.2 |
| Fish intake category 2 | 83.8±2.2 | 84.5±2.3 |
| Fish intake category 3 | 82.8±1.5 | 83.0±1.5 |
| Fish intake category 4 | 81.8±2.6 | 81.4±2.9 |
| Crude and adjusted means are reported for available data using the non-imputed data set.  ^1^ Frequency of dietary meat and fish intake are defined as follows: 1, ≤1 time per week; 2, >1 to 2 times per week; 3, > 2 times per week; 4, daily or multiple times per day.  ^2^ Semi-vegetarian and Regular meat eater diet categories were combined into one group to represent all individuals who eat meat that is not exclusively fish/seafood. | | |
